# Supplementary material for: Family-wide Structural Analysis of Human Numb-Associated Protein Kinases
Source: Structure. 2016 Mar 1;24(3):401–11. doi: 10.1016/j.str.2015.12.015 (PMC4780864; doi:10.1016/j.str.2015.12.015)
Supplement: Document S1. Figures S1–S3 [file mmc1.pdf]

Structure, Volume *24*

Supplemental Information

Family-wide Structural Analysis of Human Numb-Associated Protein Kinases

Fiona J. Sorrell, Marta Szklarz, Kamal R. Abdul Azeez, Jon M. Elkins, and Stefan Knapp

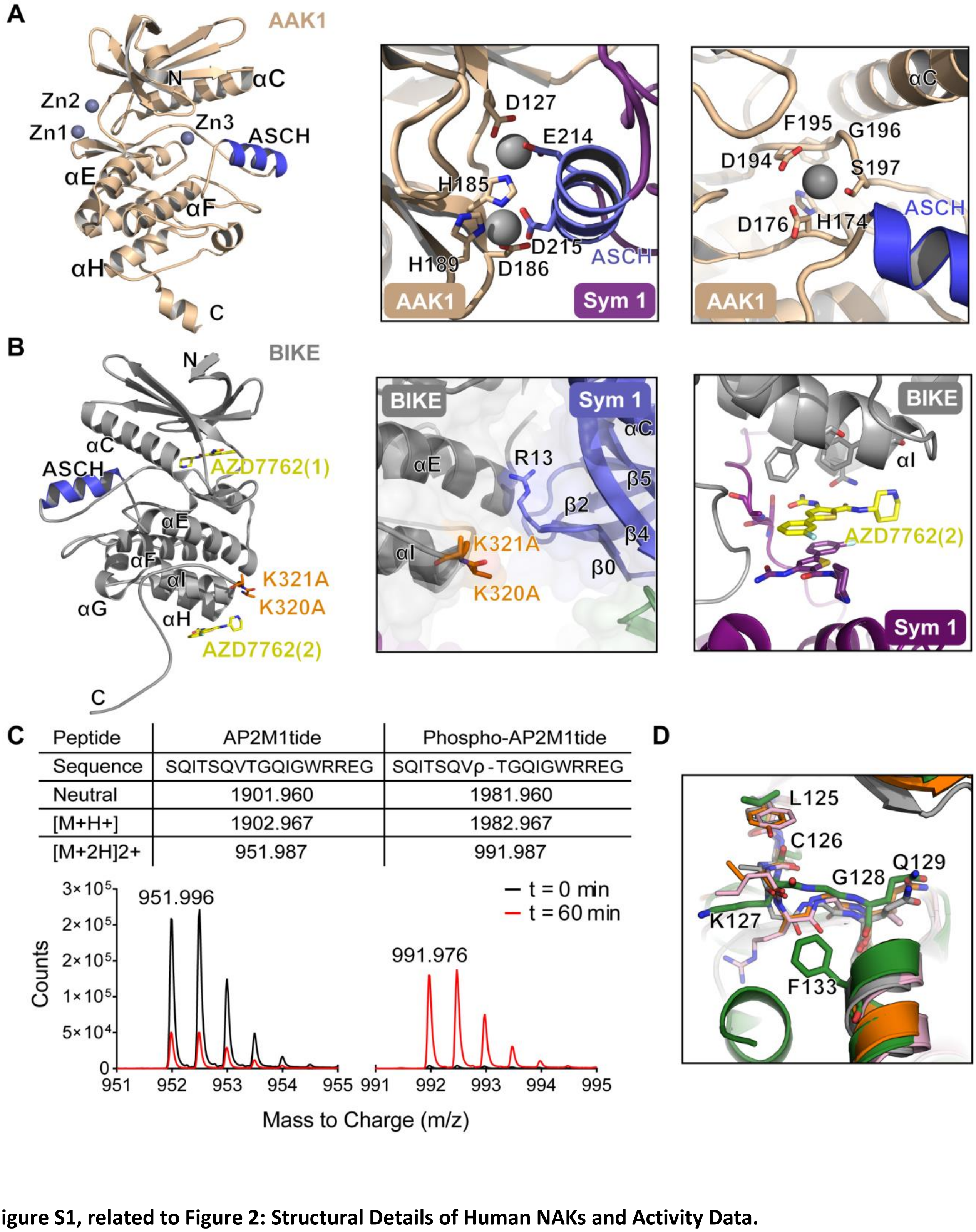

Peptide	AP2M1tide	Phospho-AP2M1tide
Sequence	SQITSQVTGQIGWRREG	SQITSQVρ-TGQIGWRREG
Neutral	1901.960	1981.960
[M+H+]	1902.967	1982.967
[M+2H]2+	951.987	991.987

Figure S1, related to Figure 2: Structural Details of Human NAKs and Activity Data.

(A) Details of AAK1 crystal structure (4wsq) including location of the activation C-terminal helix (ASCH, coloured blue), and position of zinc atoms present at high concentration in the crystallisation solution in relation to symmetry-related molecule (“Sym 1”). (B) Overview of the BIKE crystal structure (4w9w) in complex with AZD7762 showing the location of two bound ligand molecules (shown in yellow) and surface entropy

mutations (SEMs, shown in orange) used to increase crystallisability of the protein. (C) A peptide substrate "AP2M1tide" (medium subunit of AP2, residues 149-165) was phosphorylated in the presence of dephosphorylated AAK1 and the reaction monitored by mass spectrometry. Calculated expected masses are shown (top). After 1 hour in the presence of AAK1, appearance of doubly-charged phospho-peptide was observed in the mass spectrum (bottom). Similar data was obtained for BIKE. (D) Overlay of hinge region of GAK (green) with AAK1 (grey), BIKE (orange) and MPSK1 (pink) showing addition of bulky F133 in GAK that forces G128 upwards relative to other NAKs (GAK numbering).

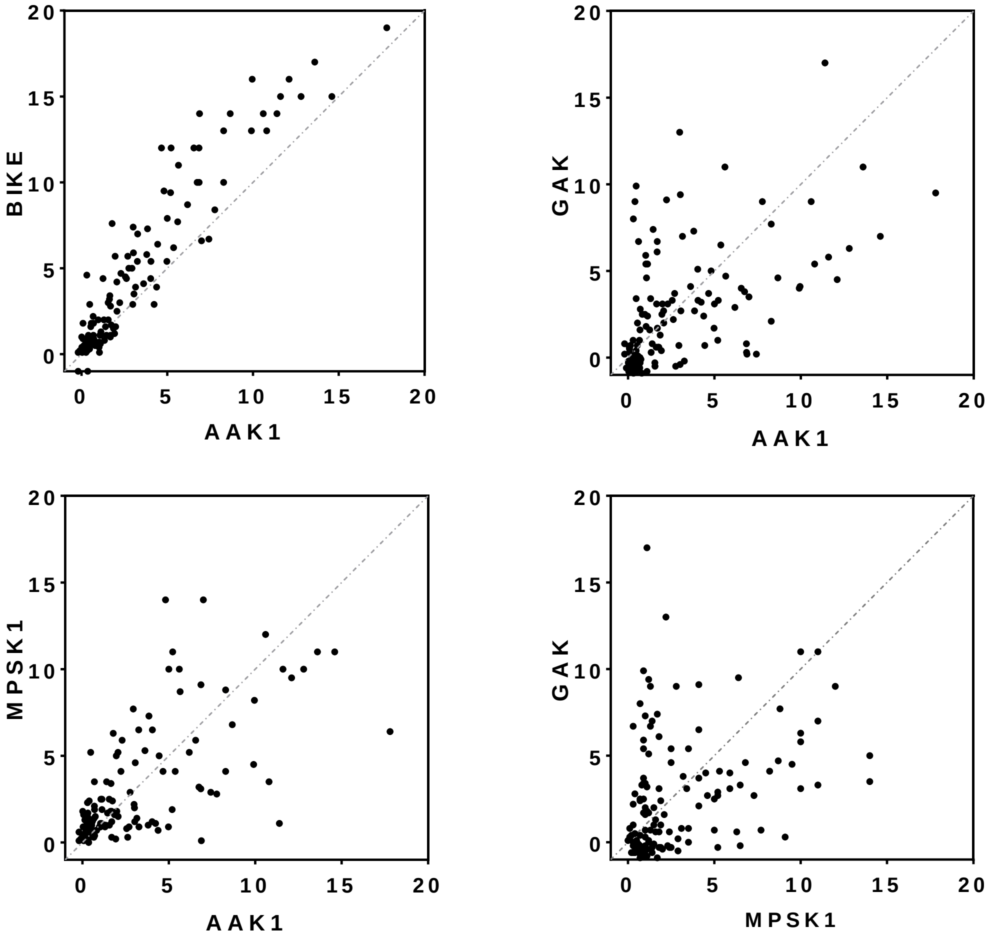

Figure S2, related to Figure 3: Thermal Shift Assay Correlation Plots for NAK Family Members.

Correlation of the measured shift in melting temperature (ΔTm, in °C) for each member of the NAK family of kinases.

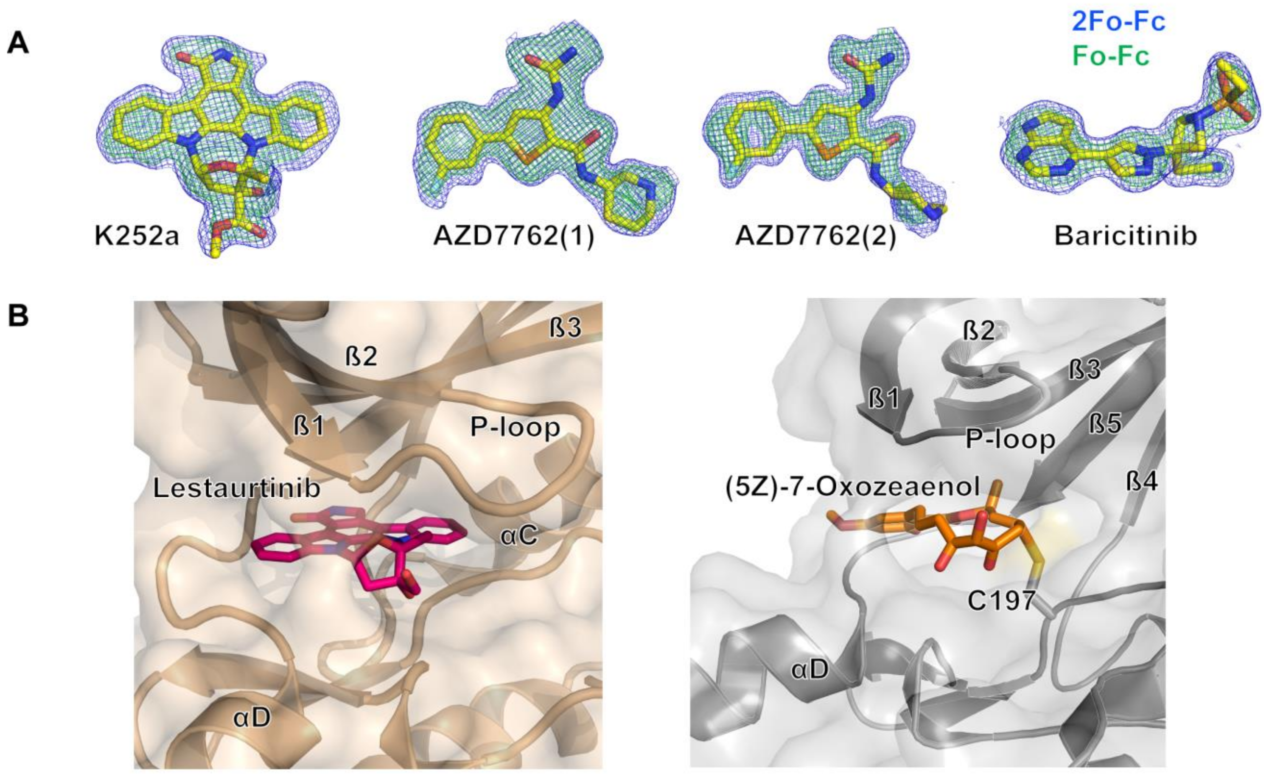

Figure S3, related to Figure 4: Ligand Binding to NAKs.

(A) Electron density omit maps for bound ligands (blue = 2Fo-Fc and green = Fo-Fc maps). K252a from pdb 4wsq, two molecules of AZD7762 from 4w9w and Baricitinib from 4w9x. (B) Models of Lestaurtinib binding to AAK1 (left) and (5Z)-7-Oxozeaenol covalent interaction with BIKE (right).

Table S1 related to figure 3.: Thermal shift assay data for clinically-approved kinase inhibitor compounds against NAK family kinases.
