# Supplementary material for: Family-wide Structural Analysis of Human Numb-Associated Protein Kinases
Source: Structure. 2016 Mar 1;24(3):401–11. doi: 10.1016/j.str.2015.12.015 (PMC4780864; doi:10.1016/j.str.2015.12.015)
Supplement: Document S2. Article plus Supplemental Information [file mmc3.pdf]

Article

Structure

Family-wide Structural Analysis of Human Numb-Associated Protein Kinases

Graphical Abstract

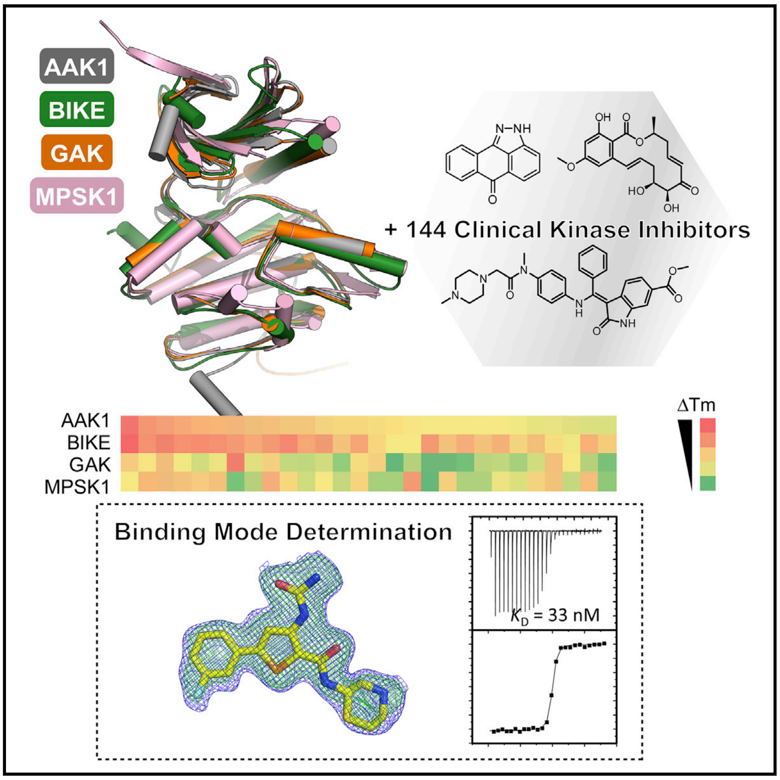

Authors

Fiona J. Sorrell, Marta Szklarz, Kamal R. Abdul Azeez, Jon M. Elkins, Stefan Knapp

Correspondence

knapp@pharmchem.uni-frankfurt.de

In Brief

The diverse Numb-associated kinases have broad cellular functions and consequently are linked with diverse-ranging diseases as well as off-target effects of clinical drugs. The first structures of AAK1 and BIKE reveal that all members of the family share unusual activation segment architecture and are highly druggable.

Highlights

- First crystal structures of AAK1 and BIKE solved, completing the NAK family
- Structural analysis of NAKs performed, revealing unusual family architecture
- 144 clinical kinase inhibitors screened against AAK1, BIKE, GAK, and MPSK1
- Nanomolar and covalent inhibitors discovered from clinical kinase library

Accession Numbers

4wsq
4w9w
4w9x

CrossMark
Sorrell et al., 2016, Structure *24*, 401–411
March 1, 2016 ©2016 The Authors
http://dx.doi.org/10.1016/j.str.2015.12.015

Family-wide Structural Analysis of Human Numb-Associated Protein Kinases

Fiona J. Sorrell,[1] Marta Szklarz,[1] Kamal R. Abdul Azeez,[1] Jon M. Elkins,[1] and Stefan Knapp[1,2,*]
[1]Nuffield Department of Clinical Medicine, Structural Genomics Consortium and Target Discovery Institute (TDI), University of Oxford, Old Road Campus, Roosevelt Drive, Oxford OX3 7DQ, UK
[2]Institute for Pharmaceutical Chemistry, Buchmann Institute for Life Sciences Campus Riedberg, Goethe-University Frankfurt, 60438 Frankfurt am Main, Germany
*Correspondence: knapp@pharmchem.uni-frankfurt.de
http://dx.doi.org/10.1016/j.str.2015.12.015
This is an open access article under the CC BY license (http://creativecommons.org/licenses/by/4.0/).

SUMMARY

The highly diverse Numb-associated kinase (NAK) family has been linked to broad cellular functions including receptor-mediated endocytosis, Notch pathway modulation, osteoblast differentiation, and dendrite morphogenesis. Consequently, NAK kinases play a key role in a diverse range of diseases from Parkinson's and prostate cancer to HIV. Due to the plasticity of this kinase family, NAK kinases are often inhibited by approved or investigational drugs and have been associated with side effects, but they are also potential drug targets. The presence of cysteine residues in some NAK family members provides the possibility for selective targeting via covalent inhibition. Here we report the first high-resolution structures of kinases AAK1 and BIKE in complex with two drug candidates. The presented data allow a comprehensive structural characterization of the NAK kinase family and provide the basis for rational design of selective NAK inhibitors.

INTRODUCTION

The Numb-associated family of protein kinases (NAKs) constitute a diverse family in terms of Ser/Thr kinases in both their function and structure, sharing little conservation outside of the kinase domain (Smythe and Ayscough, 2003) and as low as 30% sequence identity across their kinase domains. They are named for similarity to the *Drosophila* protein NAK, which plays a role during asymmetric cell division through its association with Numb. Humans have four known homologs: AAK1 (adaptor-associated kinase 1), BIKE/BMP2K (BMP-2-inducible kinase), GAK (cyclin G-associated kinase), and MPSK1 (myristoylated and palmitoylated serine/threonine kinase 1, also known as STK16).

The NAKs are associated with a broad range of cellular functions. AAK1 has a critical role in receptor-mediated endocytosis, including direct binding to clathrin and specific phosphorylation of the medium subunit of AP2 (adaptor protein 2), which is known to stimulate binding to cargo proteins (Conner and Schmid, 2002; Henderson and Conner, 2007; Neveu et al., 2012). AAK1 also modulates the Notch cell-to-cell signaling pathway by promoting Notch activation through interaction with a membrane-tethered form of Notch (Gupta-Rossi et al., 2011). Conversely, the protein Numb is thought to antagonize Notch signaling by increasing Notch degradation through polyubiquitination. Phosphorylation of Numb by AAK1 is a priming step necessary to allow its phosphorylation by other kinases (Sorensen and Conner, 2008), and thereby the role of AAK1 in the Notch pathway has been suggested to be two-fold: priming and redistribution of Numb as well as Notch activation (Gupta-Rossi et al., 2011). AAK1 is also a substrate for NDR1/2 phosphorylation and has been shown to control dendrite morphogenesis in developing mammalian neurons (Ultanir et al., 2012). BIKE is structurally closely related to AAK1 and plays a role in osteoblast differentiation, and has also recently been identified as clathrin-coated vesicle-associated protein (Borner et al., 2012), and similarly to AAK1 it is Numb associated (Krieger et al., 2013). GAK is a known association partner of cyclin G and CDK5 and among its known functions some are shared with AAK1. It is essential for clathrin trafficking and mediates binding to the plasma membrane and *trans*-Golgi network, as well as being required for maintenance of centrosome maturation and progression through mitosis (Chaikuad et al., 2014). MPSK1 is the most distantly related of the family members and its physiological functions remain poorly understood, although it is known to be a Golgi-associated kinase with a role in the regulation of secretion in the constitutive secretory pathway at the *trans*-Golgi network (In et al., 2014). In addition, MPSK1 has also been linked to mammary development in mice (Stairs et al., 2005).

Due to their wide-ranging functions, NAKs have been discussed as potential drug targets; for example, GAK has been implicated in Parkinson's, prostate cancer, and osteosarcoma (Beilina et al., 2014; Perrett et al., 2015; Sakurai et al., 2014; Susa et al., 2010; Wang et al., 2012) while BIKE has been associated with myopia (Liu et al., 2009) and AAK1 has recently been linked to a familial form of motor neuron disease known as amyotrophic lateral sclerosis (Shi et al., 2014). An interesting possible new therapeutic role for NAKs is their potential as anti-viral targets: inhibitors of AAK1 and GAK disrupt hepatitis C virus assembly (Neveu et al., 2012), and targeting BIKE has been suggested as a potential strategy for the treatment of HIV (Zhou et al., 2008). However, off-target effects have also been reported for NAK family members; for instance, potent inhibition of GAK by the cancer drug gefitinib has been

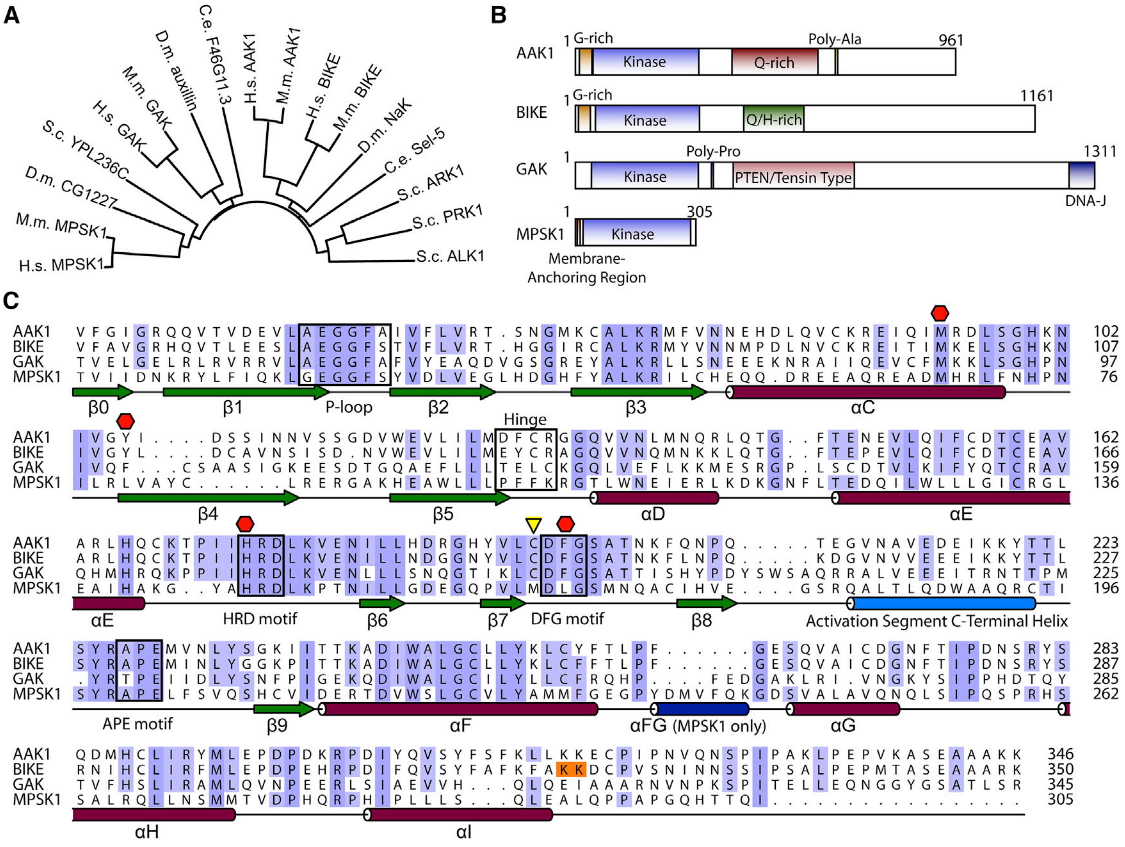

Figure 1. Sequence Conservation and Domain Organization of NAK Family

(A) NAK family phylogenetic tree. C.e., *Caenorhabditis elegans*; D.m., *Drosophila melanogaster*; H.s., *Homo sapiens*; M.m., *Mus musculus*; S.c., *Saccharomyces cerevisiae*.

(B) Domain organization of human NAKs.

(C) Sequence alignment of human NAK catalytic domains colored by residue conservation. Secondary structure as well as structural elements important for NAK function and inhibitor interactions are highlighted (green arrow, β sheet; red cylinder, α helix; blue cylinders, NAK-specific helices; yellow triangle indicates the active-site cysteine important for covalent inhibitor binding; red hexagon indicates regulatory spine residues; residues boxed in orange indicate the position of mutated residues in BIKE that have been introduced to aid crystallization).

linked to respiratory side effects in lung cancer patients (Tabara et al., 2011).

Previously we have solved the crystal structures of both MPSK1 and GAK, revealing atypical kinase activation segment architecture (Chaikuad et al., 2014; Eswaran et al., 2008). Here, we determined high-resolution crystal structures of the two remaining family members, AAK1 and BIKE, now enabling family-wide structural analysis of these interesting signaling molecules. Our analysis revealed target-specific structural features that may help to design specific chemical probes, which would help to delineate the complex functional roles of this unusual kinase family as well as provide models for our understanding of NAK off-target activity. To facilitate this, we screened a library of clinically used compounds against the kinase domain of each of the four NAK family members. Strikingly, our data show that the NAKs are able to accommodate a wide variety of ligands, suggesting excellent druggability of NAK kinases. The presented data provide a basis for rational design of selective NAK inhibitors as well as highlighting interactions of late-stage drug candidates.

RESULTS AND DISCUSSION

Phylogenetic analysis (Figure 1A) revealed that within the NAK family, hGAK and hMPSK1 cluster on separate branches of the tree along with their non-mammalian homologs, whereas the AAK1 and BIKE branches cross over due to the higher degree of sequence homology. AAK1 and BIKE are more closely related to the founding member of the family, NAK. Human MPSK1 has around 33% identity with NAK across the kinase domain. The yeast homolog ARK1 has 38% similarity to NAK in the kinase domain; it and several other yeast homologs, such as PRK1, cluster on a separate branch of the tree. We found that the placement of the kinase domain is evolutionarily conserved within the family, being situated close to the N terminus of the protein. Outside of the kinase domain, however, there is wide variation. For instance, the four human NAK family members bear only little resemblance to one another outside of the kinase domain, greatly varying in size and domain organization (Figure 1B). AAK1 and BIKE are the most closely related, with an overall sequence identity of 50%, rising to 74% across their kinase

Table 1. Crystallographic Data Collection and Refinement Statistics

Complex	AAK1 K252a	BIKE AZD7762	BIKE Baricitinib
PDB ID	4wsq	4w9w	4w9x
Data Collection			
Space group	$P2_12_12_1$	$I222$	$I222$
Cell constants: *a*, *b*, *c* (Å); α, β, γ (°)	68.6, 71.3, 183.6; 90, 90, 90	42.2, 112.7, 163.1; 90, 90, 90	42.3, 111.4, 163.8; 90, 90, 90
Resolution (Å)[a]	56.32–1.95 (2.00–1.95)	33.34–1.72 (1.75–1.72)	33.00–2.14 (2.20–2.14)
Unique observations[a]	66,564 (4,379)	41,784 (2,190)	21,713 (1,780)
Completeness (%)[a]	99.8 (100.0)	99.7 (99.9)	99.0 (99.3)
Redundancy[a]	4.7 (4.8)	6.2 (6.0)	2.6 (5.0)
R_{merge}[a]	0.10 (1.09)	0.06 (0.87)	0.10 (0.86)
Mn$I/\sigma(I)$[a]	8.3 (2.1)	15.1 (2.0)	9.6 (2.1)
$CC_{(1/2)}$[a,b]	99.7 (79.4)	99.9 (81.0)	99.3 (65.9)
Refinement			
Resolution (Å)	1.95	1.72	2.14
MR model	2buj	4wsq	4wsq
Copies in ASU	2	1	1
R_{work}, R_{free}	0.182, 0.208	0.168, 0.195	0.195, 0.233
No. of atoms	5,271	2,662	2,442
Average *B* factor (Å^2)	43.0	37.0	38.0
Rmsd (bonds) (Å)	0.013	0.017	0.018
Rmsd (angles) (°)	1.273	1.478	1.787
Crystallization	20% PEG 3350, 5 mM zinc acetate, 293 K	4 M sodium formate, 293 K	4 M sodium chloride, 0.1 M bis-Tris (pH 5.5), 293 K

ASU, asymmetric unit; rmsd, root-mean-square deviation.
[a]Values in parentheses indicate data for the highest-resolution shell.
[b]Mn(*I*) half-set correlation as reported by Aimless.

domains. GAK and MPSK1 are much more distantly related, having only 39% and 30% sequence identity, respectively, to AAK1 over their kinase domains and marginally higher sequence identity with BIKE (40% and 30%) (Figure 1C).

Family-wide Comparison Reveals Unique NAK Family Structural Features

To better understand the structural relationship across the NAK family, we solved the crystal structures of human AAK1 and BIKE. The structure of AAK1 was solved in complex with a small-molecule inhibitor to 1.95 Å resolution in space group $P2_12_12_1$ with two AAK1 kinase domains per asymmetric unit (chains A and B) (Table 1). Both chains were similar in conformation with differences only at the N and C termini (Figure 2A). In chain A, the end of the C terminus forms part of an α helix that extends outward from the kinase domain into a solvent channel in the crystal (Figure S1). The predicted secondary structure for this area agrees with our model, suggesting that the observed helix is not induced by crystal contacts. The best-diffracting crystals of AAK1 grew from a set of conditions containing a high concentration of zinc. There were several strong electron density peaks with coordination geometry and bonding distances indicative of metal ions likely representing bound zinc ions. One such electron density peak was observed at the catalytic magnesium binding site adjacent to the ATP binding pocket, indicating that zinc has replaced magnesium in this site (Figure S1). Calculation of an anomalous difference Fourier map also showed that the ions are most likely to be zinc and not magnesium, although probably not at full occupancy. Given the high zinc concentrations in the experiment, it seems unlikely that under physiological conditions zinc would occupy these sites.

More than 50 BIKE kinase domain-containing constructs were prepared, but fewer than ten were found to express a reasonable yield of soluble protein, and of these none yielded crystals. In an attempt to increase the crystallizability of one of the well-expressing constructs (BIKE residues S38–E345), six different surface-entropy-reduction (SER) mutants were prepared. In the SER constructs, up to three lysine residues on the surface of the protein were truncated to a lower-entropy alanine residue, to improve the likelihood of forming good crystal contacts (Longenecker et al., 2001). A $BIKE_{38–345}$, K320A, K321A construct gave crystals that diffracted well. Crystal structures of BIKE comprised one molecule per asymmetric unit, in space group $P2_12_12_1$, and were refined at 1.72 and 2.14 Å resolution for two different inhibitor co-crystals (Table 1 and Figure S1). The SER mutations reside near the C terminus and were designed to have little effect on the overall function of the protein, since they lie at a distant position from the ATP and substrate binding sites. Surprisingly, the mutated residues do not participate directly in any crystal-packing contacts, but it is plausible that the mutation K320A prevents electrostatic repulsion or a direct steric clash with the side chain of R13 from a symmetry neighbor, making crystallization more favorable (Figure S1).

The determination of crystal structures for AAK1 and BIKE presented here resulted in a complete structural coverage of the human NAK family. In addition, of the available GAK structures, two distinct conformations have been reported (Chaikuad et al., 2014); an inactive dimeric form where the activation segment of one monomer is exchanged with the second unit of the dimer; and an active monomeric form with fully folded activation segment, which closely resembles the activation segment architecture of MPSK1 (Eswaran et al., 2008). The structures of AAK1 and BIKE bear closest resemblance to the monomeric form of GAK (PDB: 4c57) comprising a canonical bilobal catalytic domain structure (Figure 2A). While catalytic domain structural elements are usually tightly conserved in protein kinases, the NAK family has previously been shown to diverge from usual kinase structure by the addition of a large α-helical insert, positioned C-terminal to the activation segment, named the activation segment C-terminal helix (ASCH). The activation segment of protein kinases has been termed the "most important regulatory element," whose conformation has a direct impact on both substrate interactions and catalytic efficiency (Kornev and Taylor, 2010). Our structures of AAK1 and BIKE now confirm the existence of the atypical activation segment architecture in all NAK family members (Figure 2A).

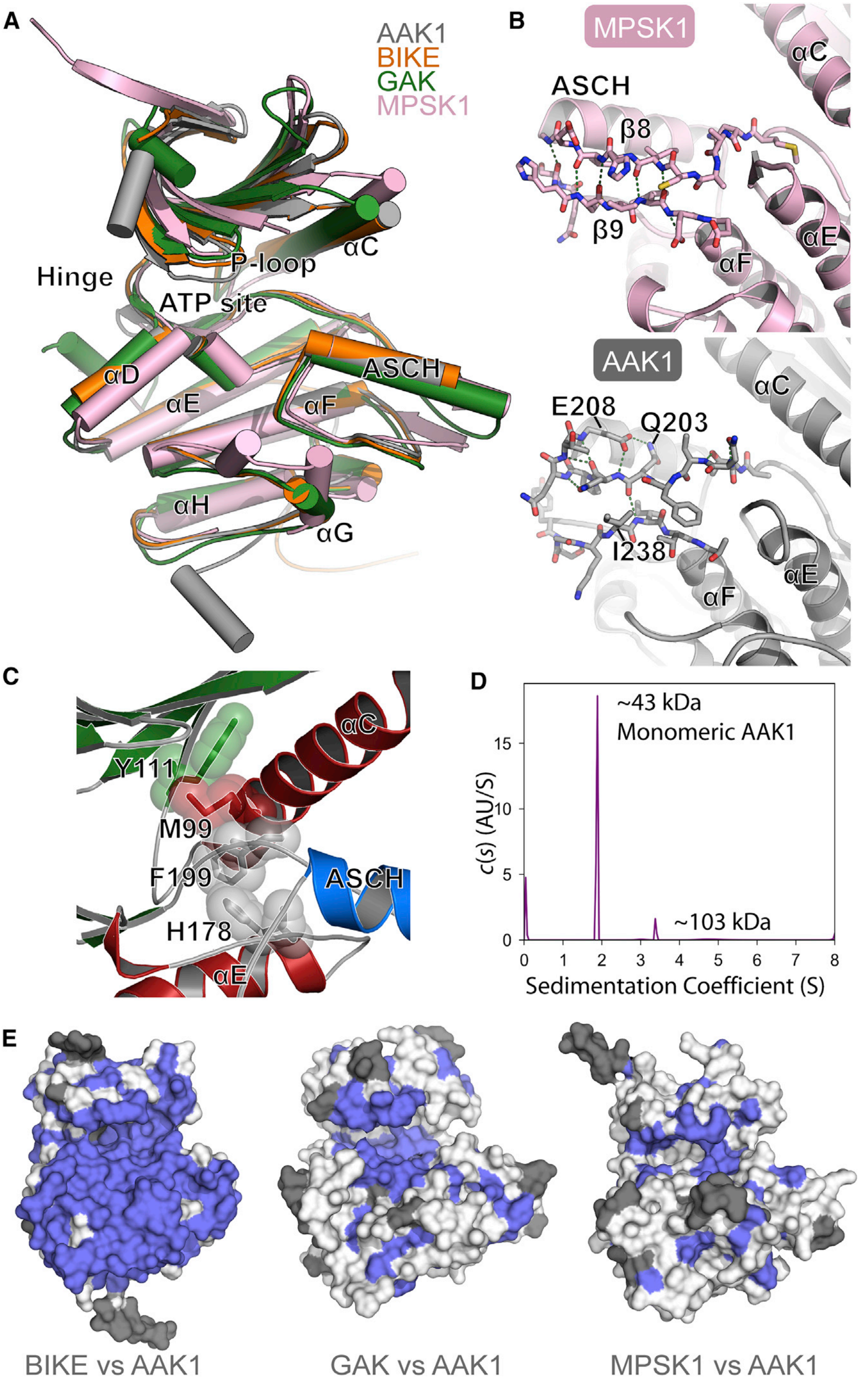

Figure 2. Crystal Structures of Human NAK Family Kinase Domains

(A) Overlay of crystal structures of AAK1 (gray, PDB: 4wsq), BIKE (orange, PDB: 4w9w), GAK (green, PDB: 4o57), and MPSK1 (pink, PDB: 2buj) showing location of the activation segment C-terminal helix (ASCH). See also Figure S1.

(B) Comparison of ASCH in MPSK1 versus AAK1.

(C) Alignment of R spine in BIKE.

(D) Sedimentation velocity of phosphorylated AAK1 showing that it is monomeric in solution.

(E) Sequence and structure alignment of NAKs, with conserved residues shown in violet, non-conserved residues in white, non-aligned residues in gray.

The ASCH in MPSK1 is positioned by an anti-parallel β-sheet segment that connects the activation segment and the αE/αF loop (Figure 2B) (Eswaran et al., 2008). In monomeric GAK, a similar array of stabilizing interactions exists across the loop. In AAK1 hydrogen bonds link residues Q203, N-terminal of the ASCH, to I239 in the αE/αF loop, and similarly in BIKE, corresponding interactions exist between L207 and I243. In general, however, the network of interactions that is observed in MPSK1 and GAK is disrupted in the crystal structures of AAK1 and BIKE (Figure 2B). Furthermore, in both AAK1 and BIKE the ASCH is shorter comprising three turns, compared with four in MPSK1 and GAK. Residues 204–208 in AAK1 (and equivalent residues in BIKE) are stabilized by a series of intramolecular interactions to form a short turn that is separated from the rest of the ASCH by a single glycine residue, which offers greater conformational freedom and results in a shorter or kinked ASCH overall. The existence of almost identical conformations of the ASCH for AAK1 and BIKE in different crystal forms suggests that this likely reflects a stable, biologically relevant conformation for substrate recognition. Other key interactions of the activation loop are conserved between all NAK family members; for instance, the glutamate side chain from the kinase Ala-Pro-Glu ("APE") motif (E229 in AAK1, Figure 1C) forms a salt bridge that secures the C terminus of the loop. Furthermore, the conserved arginine and tyrosine residues preceding the APE motif (residues Y225 and R226 in AAK1) appear to help lock the activation segment into position.

Both GAK and MPSK1 structures suggested that activation loop phosphorylation is not essential for assembly of the regulatory spine ("R spine"; residues constituting the R spine are highlighted by red hexagons in Figure 1C), leading to constitutively active proteins (Chaikuad et al., 2014; Eswaran et al., 2008; Kornev and Taylor, 2010). Similarly, in structures of BIKE and AAK1 there is alignment of the R spine in the absence of phosphorylation, suggesting that NAK family members are constitutively active kinases (Figure 2C). In AAK1, M94 from the α-C helix, Y106 from β4, and H174 and F195 from the HRD and DFG motifs, respectively, constitute the assembled R spine. The corresponding residues in BIKE (M99, Y111, H178, and F199) are also aligned. A 17-mer synthetic peptide corresponding to the AAK1/BIKE phosphorylation site on the medium subunit of AP2 was used to test kinase

activity. Phosphorylation of the substrate in the presence of AAK1 was confirmed by mass spectrometry (Figure S1). Similar data were obtained for BIKE (data not shown). In kinases that rely on activation segment phosphorylation for activity, typically the phosphorylated residue interacts with the arginine of the HRD motif to secure the activation loop and form an ordered substrate binding groove. In the structures of BIKE, AAK1, and active GAK, a salt bridge is formed between a glutamate residue in the ASCH and HRD motif arginine that mimics this interaction (residues R175 and E216 in AAK1). The rigid and anchored structure of the activation segment highlights a NAK-specific structural feature stabilizing the active state of these kinases.

Mechanisms of NAK Regulation Remain Poorly Understood

The catalytic activity of AAK1 has been shown to be stimulated by the binding and assembly of clathrin around the AP-2/AAK1 complex, resulting in increased phosphorylation of the μ2 subunit. Furthermore, clathrin was found to interact directly with the kinase domain of AAK1 (Conner et al., 2003). The mechanism for the increase in AAK1 activity in the presence of clathrin is unclear, but the crystal structure presented here indicates that the active conformation of AAK1 is not dependent on phosphorylation, since dephosphorylated AAK1 is able to form a conformation that is catalytically competent (Figure S1). It has been speculated that the substrate binding site may be blocked in the full-length protein by part of the C-terminal region of AAK1 in a pseudo-substrate type of interaction that can be removed through clathrin binding (Jackson et al., 2003). At the C terminus of AAK1, residues 846–852 correlate with the known NAK family substrate consensus sequence, and was proposed as a potential pseudo-substrate (Jackson et al., 2003). However, evidence later showed that clathrin stimulation also occurred in C-terminally truncated constructs (Conner et al., 2003). The mystery of AAK1 activation currently remains unsolved, and similarly little is understood about the mechanisms of regulation of other NAKs. In several of the GAK structures previously reported (PDB: 4o38, 4c58, 4c59), each kinase domain forms a homodimer that participates in activation segment exchange with its interaction partner. We recently suggested that this represents an inactive conformation of the catalytic domain due to the partial unfolding of the activation segment, leading to the absence of a substrate binding groove (Chaikuad et al., 2014). This dimer could represent a mechanism of GAK inactivation at high concentration, such as may occur at the *trans*-Golgi and focal adhesion sites, and thereby this could be specifically linked to GAK's function. Such a conformation has yet to be observed with other members of the NAK family. Previous studies of MPSK1 have indicated that it is monomeric (Eswaran et al., 2008), and our data so far show that AAK1 and BIKE exist predominantly as monomers (observed by gel filtration and crystallography). For some kinases phosphorylation is thought to be necessary for dimerization, for example the checkpoint kinase CHEK2 (Cai et al., 2009). Phosphorylation mapping of AAK1 grown from *Escherichia coli* showed that it is able to autophosphorylate at several sites on the activation loop (T207, S235) and various other sites across the protein (S115/S116, T144/T147, T170). We used analytical ultracentrifugation (AUC) to show that these phosphorylations appear to have little impact on the oligomerization state of the protein, since the major detectable species matched the monomeric molecular weight (Figure 2D). The minor peak visible at 103 kDa likely corresponds to minor impurities present in the sample, which were visible by SDS-PAGE (data not shown), rather than the AAK1 dimer (expected mass of 87.4 kDa).

NAK Substrate Binding Site Structure Varies in Accordance with Diverse Functional Roles

AAK1 and GAK are both known to bind to the medium subunit of AP-2, and AAK1 and BIKE are both known interaction partners of Numb. The four human NAKs are expressed across all tissue types and, given that several of their substrates overlap, it is unclear whether there is some functional redundancy between the three kinases or whether each is required for recruitment of different types of cargo (Conner and Schmid, 2003; Krieger et al., 2013; Uhlen et al., 2015; Zhang et al., 2009). If the latter is true, it can be reasoned that any differences in substrate selection and activity are most likely caused by factors other than the sequence of the substrate binding site, since AAK1 and BIKE are identical in sequence and structure across the substrate binding groove (Figure 2E). In contrast, MPSK1 has an extended loop between helices αF and αG that forms an additional helix (αFG helix) at the substrate binding site and leads to a deeper cleft. Both MPSK1 and GAK do not share a high degree of sequence similarity in the substrate binding groove with AAK1/BIKE, suggesting that these NAK family members recognize different substrates and interaction partners in agreement with their diverse biological functions.

Small Differences in NAK ATP Binding Sites Allow for Specific Inhibitors

NAK family members do not conform to the typical consensus sequence of the kinase glycine-rich loop (“P loop,” a flexible phosphate-binding loop at the ATP site; A53–A59 in AAK1): the first and third glycine in the sequence G-X_1-G-X_2-φ-G are replaced by residues with lower conformational freedom (Figure 1C). Despite this, the presence of a double-glycine motif due to addition of a glycine in position X_2 means that the loop retains a high degree of flexibility. This is demonstrated by the structures of BIKE where residues of this loop have above-average *B* factors and the protein is able to accommodate different inhibitor scaffolds through a change in loop conformation.

At the ATP binding pocket, AAK1 and BIKE differ in only three residues; two minor changes at the hinge region (D127 in AAK1 versus E131 in BIKE, and F127 versus Y132) and one change at the P loop (A58 versus S63) (Figure 1C). GAK has one residue fewer at the hinge region than other NAKs as well as the bulky aromatic side chain of F133 that sits directly below the hinge and forces the backbone of the hinge residue G128 upward, leading to the ATP pocket of GAK being fractionally more enclosed at the front (Figure S1). However, GAK compensates for this effect at the back of the ATP pocket where T123 replaces methionine or leucine in other NAK members, effectively making the pocket deeper as well as adding a polar side chain (Figure S1). AAK1, BIKE, and MPSK1 all have bulky aromatic residues on the outside of the hinge region, replaced by L125 in the equivalent position in GAK.

A

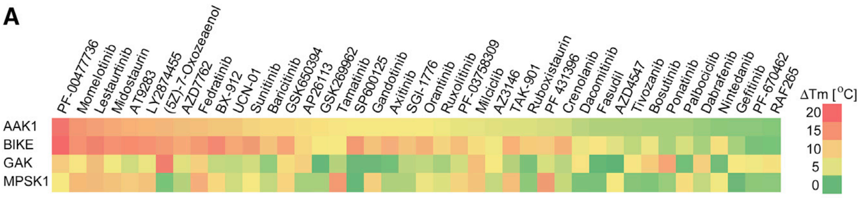

B

Compound	NAK	K_d (nM)	ΔH (cal/mol)	ΔS (cal/mol/deg)	N
AZD7762	AAK1	36.5	-1182	-7.0	1.04
	BIKE	33.1	-1485	-17.3	0.99
Baricitinib	AAK1	17.2	-1970	-33.0	1.05
	BIKE	39.8	-1541	-19.6	1.07
	GAK	134.4	-1136	-8.0	1.01
	MPSK1	68.5	-9731	-0.9	1.01
Nintedanib	AAK1	1060.4	-6320	5.4	1.03
	BIKE	104.2	-6255	10.2	1.05
AT9283	BIKE	6.0	-1056	1.0	1.07

C

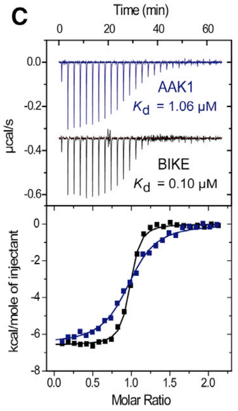

D

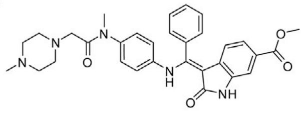

Nintedanib

E

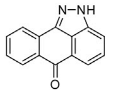

SP600125

Figure 3. Clinical Kinase Inhibitor Binding to NAKs

(A) Thermal shift assay data for a selection of clinical inhibitors against each of the NAK family kinase domains. See also Table S1 and Figure S2.

(B) ITC determination of thermodynamic parameters for inhibitor compounds determined at 15°C.

(C) ITC data measured for the interaction of nintedanib with AAK1 and BIKE. The data showed a 10-fold difference in affinity.

(D) The structure of nintedanib.

(E) The structure of SP600125.

NAKs Bind a Wide Variety of Clinical Kinase Inhibitors

To probe the ATP binding pocket of NAK family members, we screened all four kinases against a library of 144 clinically used kinase inhibitors using a thermal shift assay (Fedorov et al., 2012) (Table S1 and Figure 3A). The screening data showed a surprisingly large number of compounds with a high ΔT_m (change in protein melting temperature) for one or all NAKs, suggesting excellent druggability. Among the most significant hits were the CHK1 inhibitor PF-477736, the JAK inhibitor momelotinib, and the FLT3 inhibitor lestaurtinib. It is striking that many kinase inhibitors that have been reported to have excellent selectivity profiles in the literature interacted significantly with the NAK family. However, NAKs are often omitted from selectivity panels, including commercial screens. For instance, the JNK inhibitor SP600125 interacted strongly with BIKE and AAK1 in our assay, but showed good selectivity against 300 kinases (Anastassiadis et al., 2011); however, the panel included only MPSK1 from the NAK family (a non-binder in our assay and the most diverse member of the family). Similarly, momelotinib selectivity was profiled against approximately 150 different kinases, and of these only eight had IC_{50} < 0.1 µM; however, no NAK family members were screened (Pardanani et al., 2009). A recent study by Gao et al. (2013) profiled the binding of 158 small molecules to 234 kinases, providing an excellent overview of kinase inhibitor specificity and kinase sensitivity, but no NAKs were included in their panel. An inhibitor of the Aurora kinases and JAK2/3, AT9283, was tested against >200 kinases, but it is not clear whether NAKs were included (Dawson et al., 2010). We found that AT9283 interacted strongly with NAKs, and affinity for BIKE was confirmed by isothermal titration calorimetry (ITC) (K_D = 6 nM) (Figures 3A and 3B).

As expected from the similarity of their ATP binding sites, AAK1 and BIKE strongly interacted with the same inhibitors (Figure 3A). Correlation plots indicated very similar T_m shifts for BIKE versus AAK1, suggesting that design of selective inhibitors will be challenging (Figure S2). In contrast, GAK and MPSK1 showed significantly diverse interaction with this inhibitor set, and some inhibitors showed inhibition of only one NAK kinase. For instance, ponatinib was specific for GAK, the spleen tyrosine kinase inhibitor tamatinib interacted only with MPSK1, and the protein kinase C inhibitor UCN-01 showed preferential interaction with AAK1/BIKE. We were interested in whether the observed large values of ΔT_m correlated with high binding affinity and determined dissociation constants (K_D) for a selection using ITC. AZD7762 (ΔT_m 11°C and 13°C, respectively) had K_D = 36.5 and 33.1 nM for AAK1 and BIKE, respectively (Figure 3B). The large negative binding enthalpy change indicated favorable polar interactions. The JAK inhibitor baricitinib showed strong interaction for AAK1/BIKE in T_m assays but modest interaction with MPSK1 and GAK. ITC data confirmed strong interaction with AAK1/BIKE but revealed considerable interaction with MPSK1 and GAK despite modest T_m shifts (Figure 3B).

Crystallographic Insights into Inhibitor Selectivity

An interesting hit was nintedanib, a tyrosine kinase inhibitor in development for the treatment of idiopathic pulmonary fibrosis, which has a 10-fold higher affinity for BIKE than AAK1 (Figures 3C and 3D). The crystal structure of nintedanib in complex with vascular endothelial growth factor receptor 2 (PDB: 3c7q) revealed that the compound wraps around the outside of the hinge region. Assuming that nintedanib exploits the same type of binding mode with NAKs, it could interact with the non-conserved residues on the outside of the hinge (Figure 2E), which would explain the observed difference in binding affinities.

Differences within the ATP binding pocket may account for preferential binding of SP600125 to AAK1 and BIKE. SP600125 is a small, planar molecule (Figure 3E), and hydrophobic van der Waals interactions appear to be crucial to its binding. In crystal structures so far solved with SP600125 in complex with various kinases (PDB: 1pmv [JNK3], 1uki [JNK1], and 2zmd [TTK]), the presence of a methionine residue in the back of the ATP pocket offers a critical hydrophobic Met-π interaction with the aromatic ring. In AAK1 and BIKE, M126 and M130, respectively, are capable of fulfilling this role, but for MPSK1 and GAK the lack of methionine at this position most likely results in a weakened interaction.

To obtain insights into the molecular details of ligand interaction, we co-crystallized AAK1 with the broad-spectrum kinase

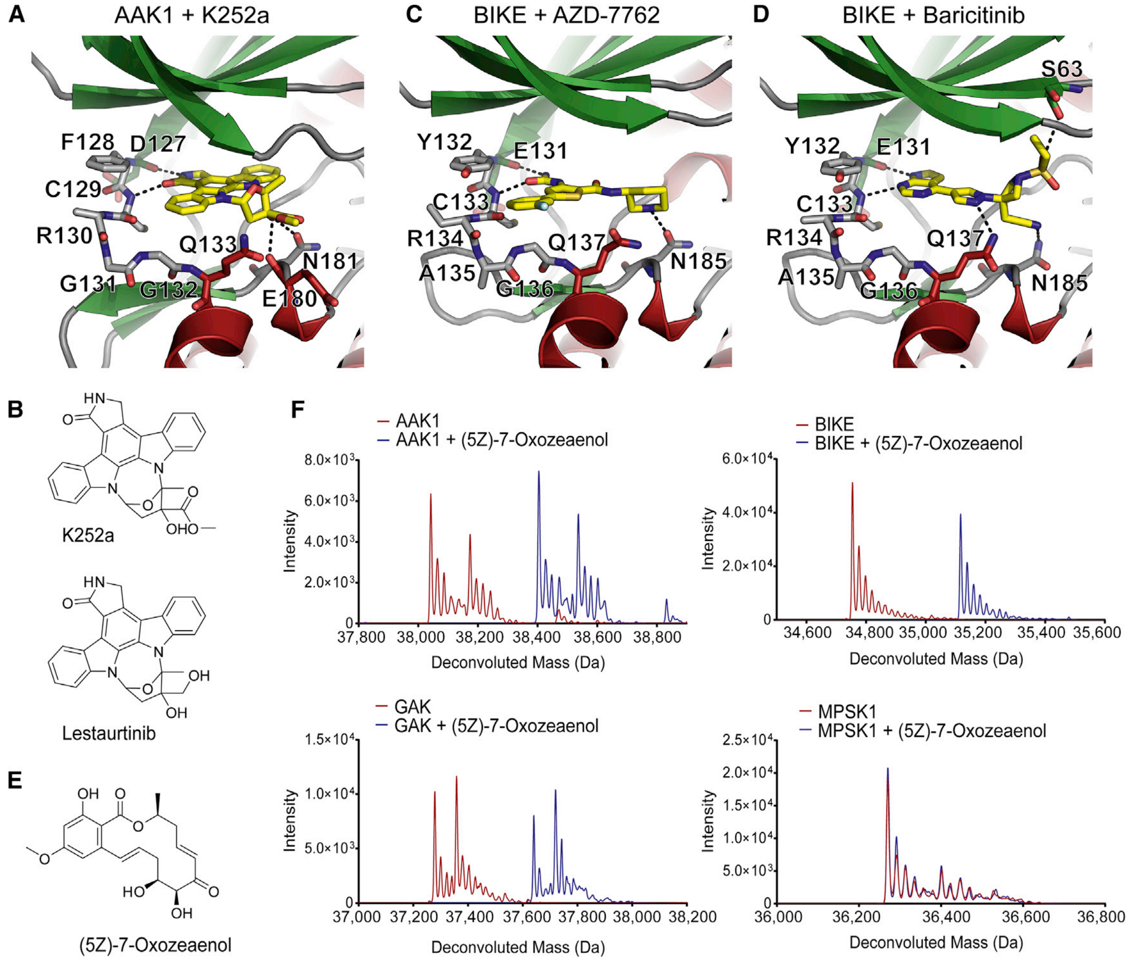

Figure 4. Small-Molecule Inhibitor Interactions with AAK1 and BIKE

(A) Interaction of K252a with AAK1. Inhibitors are shown in stick representation with yellow carbon atoms. Hydrogen bonds are indicated by dotted lines. Key interacting residues are shown and labeled.

(B) Chemical structures of K252a and related compound lestaurtinib.

(C and D) Interaction of BIKE with (C) AZD7762 with (D) baricitinib. Inhibitors are shown in stick representation with yellow carbon atoms. Hydrogen bonds are indicated by dotted lines. Key interacting residues are shown and labeled.

(E) Chemical structure of (5Z)-7-oxozeaenol.

(F) Denaturing mass spectra for NAKs in the presence of (5Z)-7-oxozeaenol.

inhibitor K252a, and BIKE with the inhibitors baricitinib and AZD7762 (Figures 4 and S3). As expected, the indolocarbazole moiety of K252a interacted with the kinase hinge, whereas the non-planar furanose tail extended out of the pocket in a similar manner to that predicted by docking of K252a into a homology model (Kuai et al., 2011). Lestautinib, closely related to K252a (Figure 4B), was found to bind with high affinity to both AAK1 and BIKE (ΔT_m of 14°C and 17°C, respectively). Based on the structure of K252a with AAK1, lestaurtinib was manually modeled in the binding pocket as shown in Figure S3.

BIKE was solved in complex with two clinically used kinase inhibitors, baricitinib and AZD7762 (Figures 4C and 4D). Baricitinib has been reported to be a potent and selective inhibitor of JAK1/2, and is currently being tested in phase III clinical trials for the treatment of rheumatoid arthritis (Norman, 2014; Shi et al., 2014; van Vollenhoven, 2013). The small molecule AZD7762, an inhibitor of the checkpoint kinases CHEK1 and CHEK2, potentiates anti-tumor activity in pre-clinical studies of various cancers when co-administered with other DNA-damage agents (Ashwell et al., 2008; Grabauskiene et al., 2014; Landau et al., 2012; Morgan et al., 2010; Zabludoff et al., 2008). Both inhibitors are ATP-competitive, forming two H-bonding interactions with the backbone of the hinge at residues E131 and C133. Baricitinib is further anchored in place by a polar interactions with Q137 and N185 at the bottom of the ATP site. The ethyl-sulfonyl group of baricitinib extends upward to interact with the P loop while, conversely, AZD7762 makes very few interactions with residues 58–62. Another notable difference between the BIKE structures is the presence of a second ligand molecule of AZD7762 positioned near the C terminus, forming π-π stacking interactions with a symmetry-related molecule (Figure S1).

ITC measurements showed about a 10-fold increased affinity of baricitinib for AAK1 and BIKE when compared with GAK (Figure 3B). The crystal structures of GAK show that F133 of

αB pushes the hinge upward, and the side chain of Q129 extends further into the ATP site relative to the corresponding residues on AAK1/BIKE, leading to non-optimal H-bonding distances with the ligand and a potential clash that could account for this difference in selectivity.

JAK inhibitors with generally good selectivity (momelotinib, AT9283, baricitinib, fedratinib, ruxolitinib, and gandotinib) interacted strongly with NAKs in our assay and, given the low similarity between these two kinase families and the diversity of ligand scaffolds involved, such extensive overlap in inhibitor activity was surprising. Unfortunately, the binding modes of JAK inhibitors with strong NAK activity have not been determined crystallographically. We can therefore only speculate that shared dynamic properties such as inherent flexibility and domain plasticity allow these kinases to accommodate similar ligand scaffolds. Ligand-interaction pattern searching (Kooistra et al., 2016) can be used to identify JAK structures with protein-ligand interactions similar to those of NAK-inhibitor complexes and estimate the binding mode. For example, in the JAK2 structure reported in PDB: 3fup, the ligand CP-690,550 shares a pyrrolopyrimidine hinge-binding motif with baricitinib, as well as a primary amine tail that extends up to make contact with the P loop of JAK2 in a similar manner to the ethyl-sulfonyl group of baricitinib in the BIKE structure we reported here. We think therefore that it is likely that the baricitinib binding mode is conserved between NAKs/JAKs.

Active-Site Cysteine Residues Allow Irreversible Inhibition by Covalent Modifiers

Other unanticipated hits in our ligand screening assay include covalent inhibitors such as (5Z)-7-oxozeaenol (Figure 4E), an inhibitor of transforming growth factor β-activated kinase 1 (TAK1) and mitogen-activated protein kinases (MAPKs) (Ohori et al., 2007; Wu et al., 2013). Irreversible inhibition of these kinases was found to be due to covalent binding of the ligand *cis*-enone moiety to a cysteine residue at the base of the ATP pocket. The crystal structures of AAK1, BIKE, and GAK reveal that these kinases possess a cysteine residue (C193, C197, and C190, respectively) at an equivalent position. In contrast, MPSK1 lacks this cysteine residue and instead a methionine (M165) is situated at this site (see Figure 1C). A high level of thermal stabilization in the presence of (5Z)-7-oxozeaenol is observed for AAK1, BIKE, and GAK, but not for MPSK1 (Figure 3A). Furthermore, when (5Z)-7-oxozeaenol was incubated with each of the NAKs, a shift in the observed molecular weight of each protein corresponding to the addition of the molecular weight of compound (a shift of around +132 Da in each case) can be observed using denaturing mass spectrometry, indicating that the compound is covalently bound except in the case of MPSK1 (Figure 4F). The kinase domain of each NAK family member possesses between 7 and 13 cysteine residues in total, several of which are located on the solvent-accessible surface of the protein. The mass spectrometry data confirm that only one inhibitor molecule is bound to each catalytic domain despite incubation with a 3-fold molar excess of covalent inhibitor and the high number of accessible cysteine residues, suggesting that the interaction is specific for the cysteine at the ATP pocket at the concentration used. Indeed, other known irreversible inhibitors such as afatinib and ibrutinib do not bind to the NAKs (Table S1). These inhibitors have been found to bind preferentially to cysteine residues located at the C-terminal region of the kinase hinge that is not conserved in NAKs. This further suggests that the covalent bond formation is specific for ligands that fit into the ATP site with a reactive group at a position complementary to the reactive side chain, and does not lead to global labeling of surface cysteine residues. We modeled (5Z)-7-oxozeaenol into the binding site of BIKE using the crystal structure of ERK2 bound to (5Z)-7-oxozeaenol (PDB: 3w55), which showed that the ligand can be neatly accommodated at this site (Figure S3).

Inhibitor Optimization and Future Perspectives

The data presented here offer a starting point for further study of these highly diverse kinases, offering potential for optimization of specific inhibitors. In BIKE, for example, it should be possible to expand into the pocket next to M130, which is occupied by a molecule of ethylene glycol and several waters in the structure with AZD7762, or by a string of water molecules in the structure with baricitinib. Despite the extraordinary similarity of the ATP binding sites of AAK1 and BIKE, by exploiting the different residues on the outside of the kinase hinge it may be possible to selectively inhibit one of these proteins. It is highly probable that this is the mechanism of the observed selective inhibition of BIKE over AAK1 by nintedanib, although further crystal structures would be required to confirm our proposed binding mode. Nevertheless, if the binding mode is indeed conserved, interaction with diverse solvent-exposed regions would offer a strategy for introducing selectivity between these closely related catalytic domains. Combining the ATP-site selectivity possibilities with similar cysteine-targeting functionality of (5Z)-7-oxozeaenol may allow development of selective covalent NAK inhibitors.

As yet, a major question remains over the redundancy of function of AAK1, BIKE, and GAK, so it is not currently clear whether there will be a need for multi-NAK-targeting ligands or whether selectivity will be key. Since NAKs appear to play a role in wide-ranging disease systems, it is indeed likely that the required specificity will be disease dependent. Development of various NAK-specific tool compounds would be a valuable addition to the future research of this family of proteins by helping to distinguish between the various functional roles of each family member and to pinpoint their specific disease contribution.

Our data show that NAKs bind with high affinity many inhibitors that were previously thought to be selective for distantly related kinases. One such example was already shown to have clinical relevance: gefitinib was originally thought to be a selective inhibitor of epidermal growth factor receptor tyrosine kinase, but was later also found to be a potent inhibitor of GAK in vivo and to cause many of the clinical effects of the drug (Sakurai et al., 2014; Tabara et al., 2011). It remains to be seen whether any of the observed effects of other clinically tested inhibitors result from off-target inhibition of members of this highly druggable branch of the kinome.

EXPERIMENTAL PROCEDURES

Expression and Purification

MPSK1$_{13–305}$ was cloned, expressed, and purified as described by Eswaran et al. (2008). AAK1$_{27–365}$ (crystallization) or AAK1$_{31–396}$ (AUC), BIKE$_{38–345(K320A,K321A)}$, and GAK$_{20–347}$ with tobacco etch virus (TEV) protease-cleavable hexahistidine tags were expressed from the vectors

pNIC-CTH0, pNIC-ZB, and pNIC28-Bsa4, respectively. Transformed BL21(DE3)-R3-pRARE cells (Novagen) were grown at 37°C in Luria-Bertani medium until OD_{600} reached 0.4–0.5, then cooled to 18°C and supplemented with 0.5 mM isopropyl 1-thio-D-galactopyranoside at an OD_{600} of 0.6 to induce protein expression overnight. Cells were harvested by centrifugation, resuspended in lysis buffer (50 mM HEPES [pH 7.5], 500 mM NaCl, 5 mM imidazole, 5% glycerol, 0.5 mM tris(2-carboxyethyl)phosphine [TCEP], 1:2,000 protease inhibitor cocktail for AAK1/BIKE; or 50 mM HEPES [pH 7.4], 500 mM NaCl, 20 mM imidazole, 5% glycerol, 0.5 mM TCEP, 0.2 mM PMSF for GAK) and lysed by sonication on ice. Proteins were purified using Ni-Sepharose resin (GE Healthcare) and eluted stepwise in binding buffer with 100–250 mM imidazole. Removal of the hexahistidine tag was performed at 4°C overnight using recombinant TEV protease either before ($AAK1_{27–365}$, BIKE) or after (GAK) gel filtration (Superdex 200 16/60, GE Healthcare). GAK and BIKE were passed over Ni-Sepharose resin as a final purification step. $AAK1_{27–365}$ was further purified using cation exchange chromatography (5-ml HiTrap Q column, GE Healthcare) to isolate the phosphorylation states. Proteins were characterized by mass spectrometry and SDS-PAGE.

Crystallization and Data Collection

Purified AAK1 or BIKE was buffer-exchanged into 50 mM HEPES (pH 7.5), 300 mM NaCl, and 5% glycerol, and concentrated to 11 or 13 mg/ml, respectively, using 30 kDa MWCO centrifugal concentrators (Millipore). Concentrated proteins were centrifuged at 14,000 rpm for 10 min at 4°C. Inhibitor compounds in DMSO were added to a final concentration of 1.5 mM (3% DMSO) and incubated on ice for approximately 30 min prior to setting up 150-nl volume sitting drops at three ratios (2:1, 1:1, or 1:2 protein-inhibitor complex to reservoir solution). Drops were equilibrated at two temperatures (4°C or 20°C). Crystals were obtained under various conditions, and were cryoprotected in mother liquor supplemented with 25% ethylene glycol before flash-freezing in liquid nitrogen for data collection. The best-diffracting crystals grew under the conditions described in Table 1. Diffraction data were collected at the Diamond Light Source beamline I04 (AAK1) or I04.1 (BIKE).

Structure Solution and Refinement

Diffraction data for both the AAK1 and BIKE crystals were integrated using MOSFLM (Leslie and Powell, 2007) and scaled using AIMLESS from the CCP4 software suite (Winn et al., 2011). For AAK1, molecular replacement was performed with Phaser (McCoy et al., 2007) using MPSK1 (PDB: 2buj) as the search model. For the two structures of BIKE, the refined AAK1 structure was used as the search model. Density modification was performed with Parrot (Zhang et al., 1997) prior to rebuilding using Buccaneer (Cowtan, 2006). Coot (Emsley et al., 2010) was used for manual model building and refinement. REFMAC5 (Murshudov et al., 1997) and PHENIX (Adams et al., 2010) were used for automated refinement, and MolProbity (Chen et al., 2010) was used to validate the structures. Structure factors and coordinates have been deposited in the PDB (see Table 1).

Sedimentation Velocity

Phosphorylated, hexahistidine-tagged $AAK1_{(31–396)}$ (molecular weight 43.7 kDa) was dialyzed into 25 mM HEPES (pH 7.5) and 150 mM NaCl, and diluted to 1.5 mg/ml (A_{280} = 1 a.u.) prior to running experiments. Data were measured at 40,000 rpm using absorbance optics in a Beckman XL-I Analytical Ultracentrifuge equipped with a Ti-50 rotor. Two sector cells were filled with sample and reference buffer. Data were analyzed using SEDFIT (Brown and Schuck, 2006) to calculate c(s) distributions.

Thermal Shift Assay

Thermal shift assay was performed according to the general procedure described by Fedorov et al. (2012). The purified catalytic domain-containing constructs of AAK1, BIKE, GAK, and MPSK1 were tested against a library of 144 kinase inhibitor compounds that have been used in the clinic (Table S1). In brief, in 96-well plate format, 19.5 μl of protein at 2 μM concentration in assay buffer (10 mM HEPES [pH 7.5], 500 mM NaCl) was incubated with SYPRO Orange dye (1:1,000 dilution) and either 0.5 μl of compound (diluted from a stock in 100% DMSO to give a final concentration of 12.5 μM compound and 2.5% DMSO) or the equivalent volume of DMSO as a reference. SYPRO Orange fluorescence intensity was measured at 25°C–95°C at a rate of 3°C/min using a real-time PCR instrument (Stratagene MxPro 3005), and ΔT_m calculated relative to an average of at least four reference wells. All measurements were performed in triplicate and mean values were reported for all compounds (Table S1).

Isothermal Titration Calorimetry

A VP-ITC or ITC_{200} (Malvern Instruments) was used to determine ligand binding affinity of various kinase inhibitors using the reverse-titration method. The concentrated kinase was dialyzed overnight in assay buffer (25 mM HEPES [pH 7.4], 150 mM NaCl, 0.5 mM TCEP) at 4°C and loaded into the ITC syringe. A stock solution of the compound at 10 or 50 mM in DMSO was diluted in dialysis buffer and added to the ITC instrument cell. Serial injections of the protein solution into the cell were made until saturation was observed. Experiments were performed at 15°C with 10–20 times the molar concentration of protein titrated into ligand. Origin software was used to analyze the data, and binding affinity was determined by fitting to a "one set of sites" model.

Protein Mass Spectrometry

A synthetic peptide substrate, residues 149–165 of the medium subunit of AP2, "AP2M1tide" (SQITSQVTGQIGWRREG), was used to test activity of dephosphorylated AAK1/BIKE. In 50 μl of total reaction volume, substrate was diluted to 100 μM in 50 mM HEPES (pH 7.5), 5 mM $MgCl_2$, 1 mM ATP, 0.5 mM TCEP, and 100 μM sodium orthovanadate. After 15 min incubation at 37°C, 250 nM purified kinase (or equivalent volume of buffer for control) was added to initiate the reaction. Reaction was performed at 37°C for 1 hr with shaking (400 rpm). 1-μl aliquots of reaction mixture were quenched after 0 and 60 min by addition of 59 μl of 0.1% formic acid and loaded onto an electrospray ionization time-of-flight (LC/MSD TOF) spectrometer (Agilent).

To test covalent inhibitor binding, we diluted purified protein to 1 mg/ml in assay buffer (25 mM HEPES [pH 7.4], 150 mM NaCl, 0.5 mM TCEP). (5Z)-7-oxozeaenol ((3*S*,5*Z*,8*S*,9*S*,11*E*)-3,4,9,10-tetrahydro-8,9,16-trihydroxy-14-methoxy-3-methyl-1*H*-2-benzoxacyclotetradecin-1,7(8*H*)-dione) (Merck) was diluted 1:10 from a 10 mM DMSO stock into assay buffer, then incubated with the protein at a final ligand concentration of 100 μM for 30 min at 4°C. 2 μl of protein was denatured by the addition of 48 μl of 1% formic acid prior to loading onto a mass spectrometer as before. The spectrum was deconvoluted using the instrument software.

Phosphorylation mapping was performed as described previously (Eswaran et al., 2008).

ACCESSION NUMBERS

The accession numbers for the data reported in this paper are PDB: 4wsq, 4w9w, 4w9x.

SUPPLEMENTAL INFORMATION

Supplemental Information includes three figures and one table and can be found with this article online at http://dx.doi.org/10.1016/j.str.2015.12.015.

AUTHOR CONTRIBUTIONS

Conceptualization, F.J.S., J.M.E., and S.K.; Investigation, F.J.S.; Writing – Original Draft, F.J.S. and S.K.; Writing – Review & Editing, F.J.S., J.M.E., and S.K.; Resources, F.J.S., M.S., and K.A.A.; Supervision, S.K. and J.M.E.

ACKNOWLEDGMENTS

We thank the Diamond Light Source for assistance with crystallographic data collection. The authors are grateful for financial support by the SGC, a registered charity (number 1097737) that receives funds from AbbVie, Bayer, Boehringer Ingelheim, the Canada Foundation for Innovation, the Canadian Institutes for Health Research, Genome Canada, GlaxoSmithKline, Janssen, Lilly Canada, the Novartis Research Foundation, the Ontario Ministry of Economic Development and Innovation, Pfizer, Takeda, and the Wellcome Trust (092809/Z/10/Z).

Received: November 4, 2015
Revised: December 18, 2015
Accepted: December 22, 2015
Published: February 4, 2016

REFERENCES

Adams, P.D., Afonine, P.V., Bunkoczi, G., Chen, V.B., Davis, I.W., Echols, N., Headd, J.J., Hung, L.-W., Kapral, G.J., Grosse-Kunstleve, R.W., et al. (2010). PHENIX: a comprehensive Python-based system for macromolecular structure solution. Acta Crystallogr. D Biol. Crystallogr. *66*, 213–221.

Anastassiadis, T., Deacon, S.W., Devarajan, K., Ma, H., and Peterson, J.R. (2011). Comprehensive assay of kinase catalytic activity reveals features of kinase inhibitor selectivity. Nat. Biotechnol. *29*, 1039–1045.

Ashwell, S., Janetka, J.W., and Zabludoff, S. (2008). Keeping checkpoint kinases in line: new selective inhibitors in clinical trials. Expert Opin. Investig. Drugs *17*, 1331–1340.

Beilina, A., Rudenko, I.N., Kaganovich, A., Civiero, L., Chau, H., Kalia, S.K., Kalia, L.V., Lobbestael, E., Chia, R., Ndukwe, K., et al. (2014). Unbiased screen for interactors of leucine-rich repeat kinase 2 supports a common pathway for sporadic and familial Parkinson disease. Proc. Natl. Acad. Sci. USA *111*, 2626–2631.

Borner, G.H., Antrobus, R., Hirst, J., Bhumbra, G.S., Kozik, P., Jackson, L.P., Sahlender, D.A., and Robinson, M.S. (2012). Multivariate proteomic profiling identifies novel accessory proteins of coated vesicles. J. Cel. Biol. *197*, 141–160.

Brown, P.H., and Schuck, P. (2006). Macromolecular size-and-shape distributions by sedimentation velocity analytical ultracentrifugation. Biophysical J. *90*, 4651–4661.

Cai, Z., Chehab, N.H., and Pavletich, N.P. (2009). Structure and activation mechanism of the CHK2 DNA damage checkpoint Kinase. Mol. Cell *35*, 818–829.

Chaikuad, A., Keates, T., Vincke, C., Kaufholz, M., Zenn, M., Zimmermann, B., Gutierrez, C., Zhang, R.G., Hatzos-Skintges, C., Joachimiak, A., et al. (2014). Structure of cyclin G-associated kinase (GAK) trapped in different conformations using nanobodies. Biochem. J. *459*, 59–69.

Chen, V.B., Arendall, W.B., III, Headd, J.J., Keedy, D.A., Immormino, R.M., Kapral, G.J., Murray, L.W., Richardson, J.S., and Richardson, D.C. (2010). MolProbity: all-atom structure validation for macromolecular crystallography. Acta Crystallogr. D Biol. Crystallogr. *66*, 12–21.

Conner, S.D., and Schmid, S.L. (2002). Identification of an adaptor-associated kinase, AAK1, as a regulator of clathrin-mediated endocytosis. J. Cel. Biol. *156*, 921–929.

Conner, S.D., and Schmid, S.L. (2003). Differential requirements for AP-2 in clathrin-mediated endocytosis. J. Cell Biol. *162*, 773–779.

Conner, S.D., Schroter, T., and Schmid, S.L. (2003). AAK1-mediated micro2 phosphorylation is stimulated by assembled clathrin. Traffic *4*, 885–890.

Cowtan, K.D. (2006). The Buccaneer software for automated model building. Acta Crystallogr. D Biol. Crystallogr. *D62*, 1002–1011.

Dawson, M.A., Curry, J.E., Barber, K., Beer, P.A., Graham, B., Lyons, J.F., Richardson, C.J., Scott, M.A., Smyth, T., Squires, M.S., et al. (2010). AT9283, a potent inhibitor of the Aurora kinases and Jak2, has therapeutic potential in myeloproliferative disorders. Br. J. Haematol. *150*, 46–57.

Emsley, P., Lohkamp, B., Scott, W.G., and Cowtan, K.D. (2010). Features and development of Coot. Acta Crystallogr. D Biol. Crystallogr. *66*, 486–501.

Eswaran, J., Bernad, A., Ligos, J.M., Guinea, B., Debreczeni, J.E., Sobott, F., Parker, S.A., Najmanovich, R., Turk, B.E., and Knapp, S. (2008). Structure of the human protein kinase MPSK1 reveals an atypical activation loop architecture. Structure *16*, 115–124.

Fedorov, O., Niesen, F.H., and Knapp, S. (2012). Kinase inhibitor selectivity profiling using differential scanning fluorimetry. Methods Mol. Biol. *795*, 109–118.

Gao, Y., Davies, S.P., Augustin, M., Woodward, A., Patel, U.A., Kovelman, R., and Harvey, K.J. (2013). A broad activity screen in support of a chemogenomic map for kinase signalling research and drug discovery. Biochem. J. *451*, 313–328.

Grabauskiene, S., Bergeron, E.J., Chen, G., Thomas, D.G., Giordano, T.J., Beer, D.G., Morgan, M.A., and Reddy, R.M. (2014). Checkpoint kinase 1 protein expression indicates sensitization to therapy by checkpoint kinase 1 inhibition in non-small cell lung cancer. J. Surg. Res. *187*, 6–13.

Gupta-Rossi, N., Ortica, S., Meas-Yedid, V., Heuss, S., Moretti, J., Olivo-Marin, J.C., and Israel, A. (2011). The adaptor-associated kinase 1, AAK1, is a positive regulator of the Notch pathway. J. Biol. Chem. *286*, 18720–18730.

Henderson, D.M., and Conner, S.D. (2007). A novel AAK1 splice variant functions at multiple steps of the endocytic pathway. Mol. Biol. Cel. *18*, 2698–2706.

In, J.G., Striz, A.C., Bernad, A., and Tuma, P.L. (2014). Serine/threonine kinase 16 and MAL2 regulate constitutive secretion of soluble cargo in hepatic cells. Biochem. J. *463*, 201–213.

Jackson, A.P., Flett, A., Smythe, C., Hufton, L., Wettey, F.R., and Smythe, E. (2003). Clathrin promotes incorporation of cargo into coated pits by activation of the AP2 adaptor micro2 kinase. J. Cell Biol. *163*, 231–236.

Kooistra, A.J., Kanev, G.K., van Linden, O.P.J., Leurs, R., de Esch, I.J.P., and de Graaf, C. (2016). KLIFS: a structural kinase-ligand interaction database. Nucleic Acids Res. *44*, D365–D371.

Kornev, A.P., and Taylor, S.S. (2010). Defining the conserved internal architecture of a protein kinase. Biochim. Biophys. Acta *1804*, 440–444.

Krieger, J.R., Taylor, P., Gajadhar, A.S., Guha, A., Moran, M.F., and McGlade, C.J. (2013). Identification and selected reaction monitoring (SRM) quantification of endocytosis factors associated with Numb. Mol. Cell. Proteomics *12*, 499–514.

Kuai, L., Ong, S.E., Madison, J.M., Wang, X., Duvall, J.R., Lewis, T.A., Luce, C.J., Conner, S.D., Pearlman, D.A., Wood, J.L., et al. (2011). AAK1 identified as an inhibitor of neuregulin-1/ErbB4-dependent neurotrophic factor signaling using integrative chemical genomics and proteomics. Chem. Biol. *18*, 891–906.

Landau, H.J., McNeely, S.C., Nair, J.S., Comenzo, R.L., Asai, T., Friedman, H., Jhanwar, S.C., Nimer, S.D., and Schwartz, G.K. (2012). The checkpoint kinase inhibitor AZD7762 potentiates chemotherapy-induced apoptosis of p53-mutated multiple myeloma cells. Mol. Cancer Ther. *11*, 1781–1788.

Leslie, A.G.W., and Powell, H.R. (2007). Processing diffraction data with mosflm. Evolving Methods Macromol. Crystallogr. *245*, 41–51.

Liu, H.P., Lin, Y.J., Lin, W.Y., Wan, L., Sheu, J.J., Lin, H.J., Tsai, Y., Tsai, C.H., and Tsai, F.J. (2009). A novel genetic variant of BMP2K contributes to high myopia. J. Clin. Lab. Anal. *23*, 362–367.

Longenecker, K.L., Garrard, S.M., Sheffield, P.J., and Derewenda, Z.S. (2001). Protein crystallization by rational mutagenesis of surface residues: Lys to Ala mutations promote crystallization of RhoGDI. Acta Crystallogr. D Biol. Crystallogr. *57*, 679–688.

McCoy, A.J., Grosse-Kunstleve, R.W., Adams, P.D., Winn, M.D., Storoni, L.C., and Read, R.J. (2007). Phaser crystallographic software. J. Appl. Crystallogr. *40*, 658–674.

Morgan, M.A., Parsels, L.A., Zhao, L., Parsels, J.D., Davis, M.A., Hassan, M.C., Arumugarajah, S., Hylander-Gans, L., Morosini, D., Simeone, D.M., et al. (2010). Mechanism of radiosensitization by the Chk1/2 inhibitor AZD7762 involves abrogation of the G2 checkpoint and inhibition of homologous recombinational DNA repair. Cancer Res. *70*, 4972–4981.

Murshudov, G.N., Vagin, A., and Dodson, E.J. (1997). Refinement of macromolecular structures by the maximum-likelihood method. Acta Crystallogr. D Biol. Crystallogr. *53*, 240–255.

Neveu, G., Barouch-Bentov, R., Ziv-Av, A., Gerber, D., Jacob, Y., and Einav, S. (2012). Identification and targeting of an interaction between a tyrosine motif within hepatitis C virus core protein and AP2M1 essential for viral assembly. PLoS Pathog. *8*, e1002845.

Norman, P. (2014). Selective JAK inhibitors in development for rheumatoid arthritis. Expert Opin. Investig. Drugs *23*, 1067–1077.

Ohori, M., Kinoshita, T., Yoshimura, S., Warizaya, M., Nakajima, H., and Miyake, H. (2007). Role of a cysteine residue in the active site of ERK and the MAPKK family. Biochem. Biophys. Res. Commun. *353*, 633–637.

Pardanani, A., Lasho, T., Smith, G., Burns, C.J., Fantino, E., and Tefferi, A. (2009). CYT387, a selective JAK1/JAK2 inhibitor: in vitro assessment of kinase selectivity and preclinical studies using cell lines and primary cells from polycythemia vera patients. Leukemia *23*, 1441–1445.

Perrett, R.M., Alexopoulou, Z., and Tofaris, G.K. (2015). The endosomal pathway in Parkinson's disease. Mol. Cell. Neurosci. *66*, 21–28.

Sakurai, M.A., Ozaki, Y., Okuzaki, D., Naito, Y., Sasakura, T., Okamoto, A., Tabara, H., Inoue, T., Hagiyama, M., Ito, A., et al. (2014). Gefitinib and luteolin cause growth arrest of human prostate cancer PC-3 cells via inhibition of cyclin G-associated kinase and induction of miR-630. PLoS One *9*, e100124.

Shi, B., Conner, S.D., and Liu, J. (2014). Dysfunction of endocytic kinase AAK1 in ALS. Int. J. Mol. Sci. *15*, 22918–22932.

Smythe, E., and Ayscough, K.R. (2003). The Ark1/Prk1 family of protein kinases. Regulators of endocytosis and the actin skeleton. EMBO Rep. *4*, 246–251.

Sorensen, E.B., and Conner, S.D. (2008). AAK1 regulates Numb function at an early step in clathrin-mediated endocytosis. Traffic (Copenhagen, Denmark) *9*, 1791–1800.

Stairs, D.B., Notarfrancesco, K.L., and Chodosh, L.A. (2005). The serine/threonine kinase, Krct, affects endbud morphogenesis during murine mammary gland development. Transgenic Res. *14*, 919–940.

Susa, M., Choy, E., Liu, X., Schwab, J., Hornicek, F.J., Mankin, H., and Duan, Z. (2010). Cyclin G-associated kinase is necessary for osteosarcoma cell proliferation and receptor trafficking. Mol. Cancer Ther. *9*, 3342–3350.

Tabara, H., Naito, Y., Ito, A., Katsuma, A., Sakurai, M.A., Ohno, S., Shimizu, H., Yabuta, N., and Nojima, H. (2011). Neonatal lethality in knockout mice expressing the kinase-dead form of the gefitinib target GAK is caused by pulmonary dysfunction. PLoS One *6*, e26034.

Uhlen, M., Fagerberg, L., Hallstrom, B.M., Lindskog, C., Oksvold, P., Mardinoglu, A., Sivertsson, A., Kampf, C., Sjostedt, E., Asplund, A., et al. (2015). Proteomics. Tissue-based map of the human proteome. Science *347*, 1260419.

Ultanir, S.K., Hertz, N.T., Li, G., Ge, W.P., Burlingame, A.L., Pleasure, S.J., Shokat, K.M., Jan, L.Y., and Jan, Y.N. (2012). Chemical genetic identification of NDR1/2 kinase substrates AAK1 and Rabin8 uncovers their roles in dendrite arborization and spine development. Neuron *73*, 1127–1142.

van Vollenhoven, R.F. (2013). Small molecular compounds in development for rheumatoid arthritis. Curr. Opin. Rheumatol. *25*, 391–397.

Wang, G., Pan, J., and Chen, S.D. (2012). Kinases and kinase signaling pathways: potential therapeutic targets in Parkinson's disease. Prog. Neurobiol. *98*, 207–221.

Winn, M.D., Ballard, C.C., Cowtan, K.D., Dodson, E.J., Emsley, P., Evans, P.R., Keegan, R.M., Krissinel, E.B., Leslie, A.G.W., McCoy, A., et al. (2011). Overview of the CCP4 suite and current developments. Acta Crystallogr. D Biol. Crystallogr. *67*, 235–242.

Wu, J., Powell, F., Larsen, N.A., Lai, Z., Byth, K.F., Read, J., Gu, R.F., Roth, M., Toader, D., Saeh, J.C., et al. (2013). Mechanism and in vitro pharmacology of TAK1 inhibition by (5Z)-7-oxozeaenol. ACS Chem. Biol. *8*, 643–650.

Zabludoff, S.D., Deng, C., Grondine, M.R., Sheehy, A.M., Ashwell, S., Caleb, B.L., Green, S., Haye, H.R., Horn, C.L., Janetka, J.W., et al. (2008). AZD7762, a novel checkpoint kinase inhibitor, drives checkpoint abrogation and potentiates DNA-targeted therapies. Mol. Cancer Ther. *7*, 2955–2966.

Zhang, K.Y., Cowtan, K.D., and Main, P. (1997). Combining constraints for electron-density modification. Methods Enzymol. *277*, 53–64.

Zhang, J., Yang, P.L., and Gray, N.S. (2009). Targeting cancer with small molecule kinase inhibitors. Nat. Rev. Cancer *9*, 28–39.

Zhou, H., Xu, M., Huang, Q., Gates, A.T., Zhang, X.D., Castle, J.C., Stec, E., Ferrer, M., Strulovici, B., Hazuda, D.J., et al. (2008). Genome-scale RNAi screen for host factors required for HIV replication. Cell Host Microbe *4*, 495–504.

Structure, Volume *24*

Supplemental Information

Family-wide Structural Analysis of Human Numb-Associated Protein Kinases

Fiona J. Sorrell, Marta Szklarz, Kamal R. Abdul Azeez, Jon M. Elkins, and Stefan Knapp

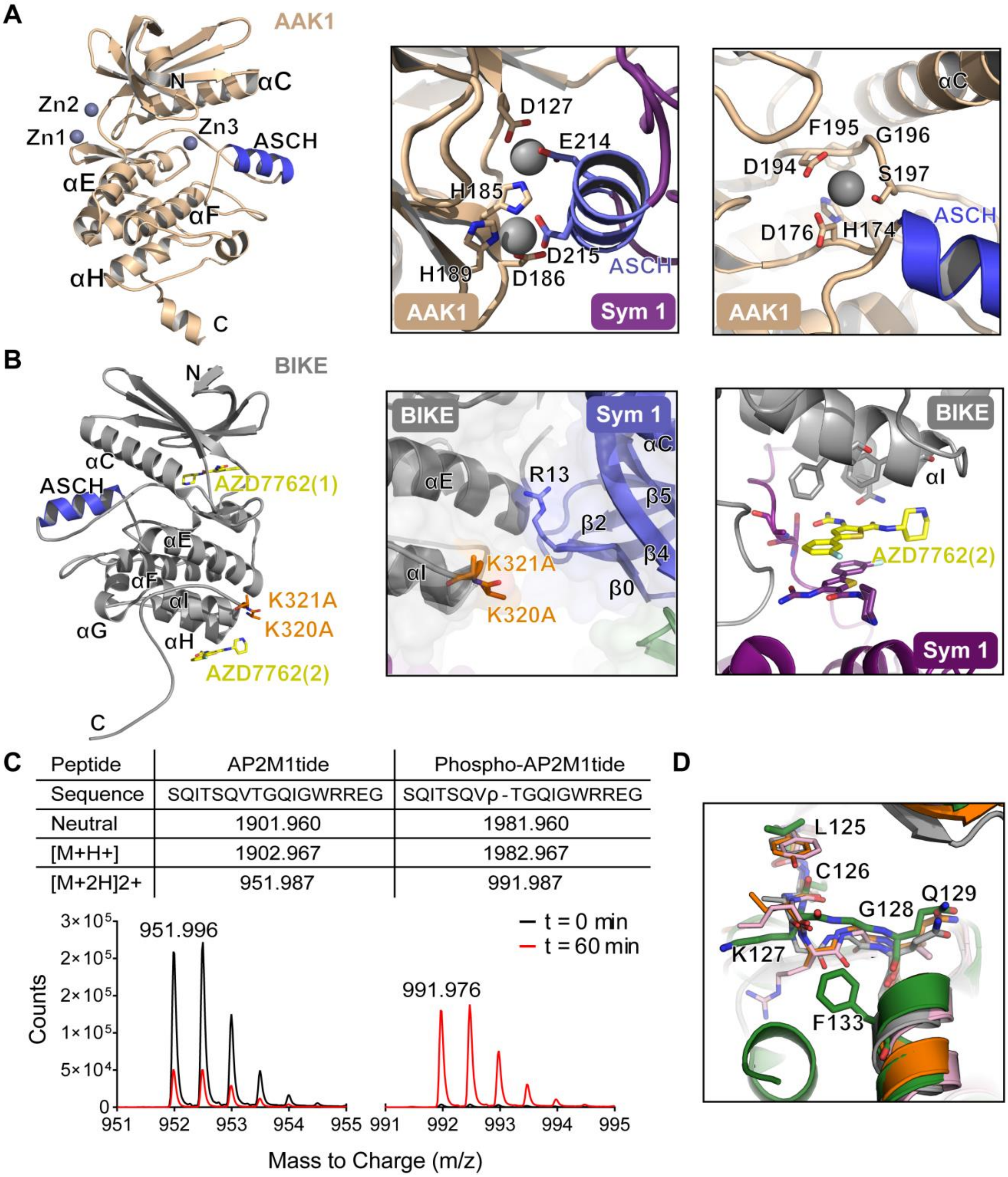

Peptide	AP2M1tide	Phospho-AP2M1tide
Sequence	SQITSQVTGQIGWRREG	SQITSQVp-TGQIGWRREG
Neutral	1901.960	1981.960
[M+H+]	1902.967	1982.967
[M+2H]2+	951.987	991.987

Figure S1, related to Figure 2: Structural Details of Human NAKs and Activity Data.

(A) Details of AAK1 crystal structure (4wsq) including location of the activation C-terminal helix (ASCH, coloured blue), and position of zinc atoms present at high concentration in the crystallisation solution in relation to symmetry-related molecule ("Sym 1"). (B) Overview of the BIKE crystal structure (4w9w) in complex with AZD7762 showing the location of two bound ligand molecules (shown in yellow) and surface entropy

mutations (SEMs, shown in orange) used to increase crystallisability of the protein. (C) A peptide substrate "AP2M1tide" (medium subunit of AP2, residues 149-165) was phosphorylated in the presence of dephosphorylated AAK1 and the reaction monitored by mass spectrometry. Calculated expected masses are shown (top). After 1 hour in the presence of AAK1, appearance of doubly-charged phospho-peptide was observed in the mass spectrum (bottom). Similar data was obtained for BIKE. (D) Overlay of hinge region of GAK (green) with AAK1 (grey), BIKE (orange) and MPSK1 (pink) showing addition of bulky F133 in GAK that forces G128 upwards relative to other NAKs (GAK numbering).

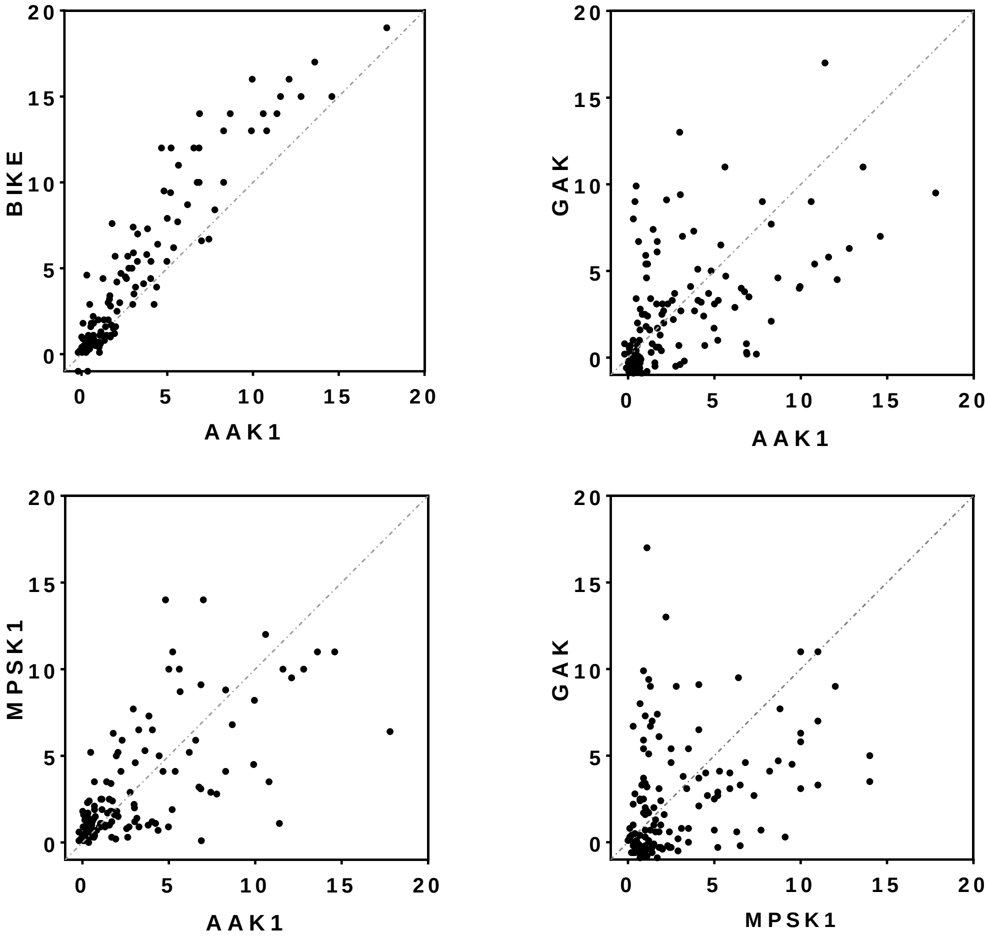

Figure S2, related to Figure 3: Thermal Shift Assay Correlation Plots for NAK Family Members.

Correlation of the measured shift in melting temperature (ΔTm, in °C) for each member of the NAK family of kinases.

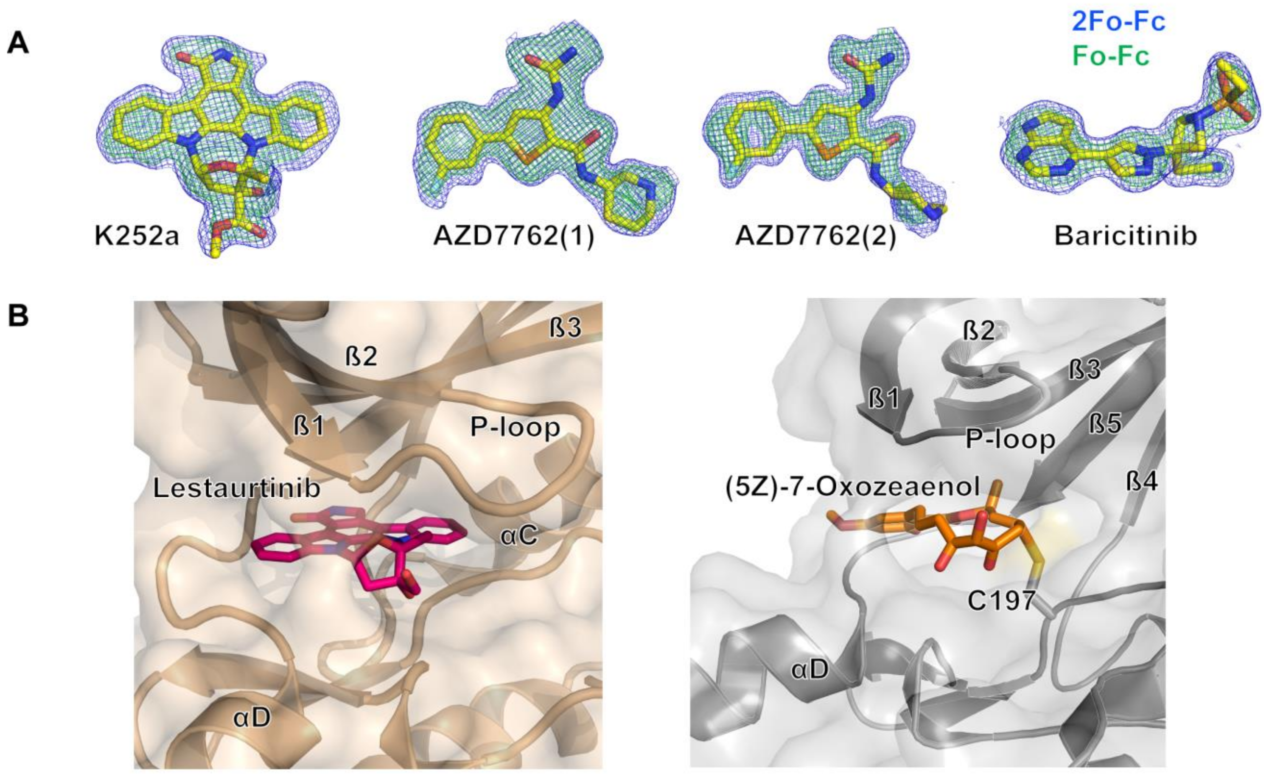

Figure S3, related to Figure 4: Ligand Binding to NAKs.

(A) Electron density omit maps for bound ligands (blue = 2Fo-Fc and green = Fo-Fc maps). K252a from pdb 4wsq, two molecules of AZD7762 from 4w9w and Baricitinib from 4w9x. (B) Models of Lestaurtinib binding to AAK1 (left) and (5Z)-7-Oxozeaenol covalent interaction with BIKE (right).

Table S1 related to figure 3.: Thermal shift assay data for clinically-approved kinase inhibitor compounds against NAK family kinases.
